# O-12 - INFECTION PREVENTION AND CONTROL FOR RESPIRATORY VIRAL INFECTIONS IN NHS TRUSTS: A NATIONAL SURVEY

**DOI:** 10.1093/pubmed/fdag046.012

**Published:** 2026-07-09

**Authors:** Dr Clare Foster, Dr Dale Weston, Dr Laura Maynard-Smith, Dr Emma McGuire, Dr Jo Taylor-Egbeyemi, Dr Holly Carter, Dr Carole Fry, Dr Lisa Ritchie, Dr Mark Wilcox, Prof Jacqui Reilly, Dr Colin Brown, Dr Ashley Sharp

**Affiliations:** UK Health Security Agency Yorkshire and the Humber Health Protection Team; Behavioural Science and Insights Unit, UK Health Security Agency; Antimicrobial Resistance & Healthcare Associated Infections Division, UK Health Security Agency; Antimicrobial Resistance & Healthcare Associated Infections Division, UK Health Security Agency; Behavioural Science and Insights Unit, UK Health Security Agency; Behavioural Science and Insights Unit, UK Health Security Agency; Antimicrobial Resistance & Healthcare Associated Infections Division, UK Health Security Agency; NHS England; NHS England; Glasgow Caledonian University; Antimicrobial Resistance & Healthcare Associated Infections Division, UK Health Security Agency; Antimicrobial Resistance & Healthcare Associated Infections Division, UK Health Security Agency

## Abstract

**Background:**

Transmission of respiratory viral infections (RVIs) in healthcare settings presents a significant risk to staff, patients and visitors. Infection prevention and control (IPC) measures, such as the use of fluid-resistant surgical masks (FRSMs) and filtering facepiece protection level 3 (FFP3) respirator masks, can be used by staff to reduce risk. In England, IPC guidance for healthcare staff is produced nationally and applied locally by NHS hospital trusts, who may seek advice from frontline health protection specialists.

**Objectives:**

Our aim was to assess how NHS hospital trusts were implementing IPC guidance for RVIs, to explore variation in the use of FRSMs and FFP3 respirators by staff, and to understand which tools were being used for workplace and individual staff risk assessments.

**Methods:**

A cross-sectional online survey of NHS trusts in England was conducted in February-March 2023. Responses were analysed using Fisher's exact tests and the framework approach.

**Results:**

Responses were received from 59% of eligible hospital trusts. All trusts required staff to wear facemasks or respirators when providing direct care to patients with suspected/confirmed respiratory viral infections. However, there was substantial variation in the type of facemask/respirator required in different settings and scenarios. Over half of the trusts relied on locally developed risk assessment tools, leading to significant variation in mask use policies. The survey also highlighted variations in fit testing policies and compliance monitoring.

**Conclusions:**

There was marked variation in the implementation of IPC guidance and in the use of workplace and individual risk assessments across NHS hospital trusts. Whilst local flexibility enables adaptation to local contexts, it may also result in inconsistent protection for healthcare staff. Greater standardisation or clearer nationally available tools may support more consistent and equitable IPC practice across settings.

